# Copper metabolism patterns and tumor microenvironment characterization in colon adenocarcinoma

**DOI:** 10.3389/fonc.2022.959273

**Published:** 2022-09-20

**Authors:** Jianwei Lin, Bixian Luo, Xinbo Yu, Zheyu Yang, Mingliang Wang, Wei Cai

**Affiliations:** ^1^Department of General Surgery, Ruijin Hospital, Shanghai Jiao Tong University School of Medicine, Shanghai, China; ^2^Department of Urology, Ruijin Hospital, Shanghai Jiao Tong University School of Medicine, Shanghai, China; ^3^Department of General Surgery, Ruijin Hospital Luwan Branch, Shanghai Jiao Tong University School of Medicine, Shanghai, China

**Keywords:** copper metabolism, microenvironment, colon adenocarcinoma (COAD), risk score, nomogram

## Abstract

Copper participates in biological processes such as oxygen metabolism and iron uptake, and is a key factor in immune regulation. Based on the transcription data, mutation data and clinical data of colon adenocarcinoma (COAD) patients from The Cancer Genome Atlas (TCGA) database and Gene Expression Profiling Interactive Analysis (GEPIA2) database, the expression and mutation of copper metabolization-related genes in COAD patients and their correlation with tumor immune microenvironment were analyzed. Copper metabolization-related genes (CMRGs) were used to construct COAD subtypes and prognostic risk models for COAD patients. Furthermore, Kaplan-Meier (K-M) curve and receiver operating characteristic (ROC) curve were used to analyze the clinical value of COAD subtypes and genotyping models in distinguishing clinical characteristics of patients, and the immune infiltration of patients with different genotypes was analyzed. Finally, the clinical tissue samples from COAD patients were used to analyze the mRNA expression of genes in risk model between tumor and normal tissues by the method of Polymerase Chain Reaction (PCR). Of the 479 CMRGs, 68 genes were differentially expressed in normal and tumor tissues of COAD patients in TCGA and GEPIA2. Two subtypes with different clinical and immunological characteristics were identified by using 482 genes related to copper metabolism. Finally, a prognostic risk model consisting of five CMRGs was constructed, which could not only predict the prognosis of patients, but also correlated with COAD subtypes. In addition, some genes (glutathione S-transferase mu 1, cyclin D1and cytochrome P450 family 2 subfamily S member 1) in risk model was show significant difference between normal and tumor tissues. The COAD subtypes identified by CMRGs can help clinically distinguish patients with different prognosis and tumor progression, and the risk score can assist in clinical evaluation of patient prognosis, serving as a valuable biomarker for COAD immunotherapy.

## Introduction

Colon cancer is the fifth most common malignancy worldwide and the fifth leading cause of death in cancer patients (1). Adenocarcinoma of the colon is the most common tumor type of colon cancer (2). In recent years, the age of onset of colon adenocarcinoma (COAD) has become younger and more aggressive (3). However, the current TNM staging used to assess patients’ prognosis and disease development status has certain limitations in clinical use, resulting in differences in treatment outcomes for patients (4, 5). In addition, due to tumor recurrence, metastasis, and other reasons, patients often lead to treatment failure (6). Therefore, it is necessary to find more biomarkers with clinical application value and therapeutic significance to improve the survival and prognosis of COAD patients.

Copper is an important metal element involved in oxygen metabolism, iron uptake and other biological functions in cells (7). Abnormal copper metabolism will not only lead to hereditary neurodegenerative diseases, but also lead to mitochondrial damage in normal cells, thus affecting cell growth (8–10). Recent studies have shown that copper accumulation triggers the aggregation of mitochondrial lipoylated proteins and the destabilization of Fe–S cluster proteins, leading to a unique type of cell death termed cuproptosis (11). Copper metabolism, which can lead to mitochondrial damage by affecting mitochondrial metabolism and mitochondrial function, may be involved in the process of mitochondrial influence on tumor. Therefore, copper metabolization-related genes (CMRGs) can become potential targets for tumor therapy and important biomolecular markers for predicting tumor prognosis.

In this study, the expression of 479 CMRGs were analyzed using TCGA database and GEPIA2 database, and the patterns of CMRGs and characteristics of tumor immune microenvironment in COAD patients were systematically evaluated. Meanwhile, two COAD subtypes with different characteristics were identified by CMRGs, and survival differences and clinical features within the same subtype were analyzed. Classification of subtypes and prognostic models may be an effective means to evaluate the treatment of COAD patients, and CMRGs may be an important biomolecular target for immunotherapy of COAD.

## Materials and methods

### Data collection

The mRNA expression data, clinical information and mutant data downloaded from TCGA database (https://portal.gdc.cancer.gov/repository) were used to analyze the expression of copper metabolization-related genes, expression patterns and characteristics of tumor immune microenvironment in COAD patients, and to construct risk model. The database contains 39 normal samples and 398 tumor samples. Data set GSE39582, downloaded from the GEO database(http://www.ncbi.nlm.nih.gov/geo/) containing mRNA expression data and clinical information from tumor tissues of 585 COAD patients, was used to validate the risk model. The platform of GSE39582 is [HG-U133_Plus_2] Affymetrix Human Genome U133 Plus 2.0 Array. CMRGs download from GeneCards database (https://www.genecards.org/). According to correlation score > 7, a total of 482 protein-coding genes related to copper metabolism were collected for screening, among which synthesis of cytochrome C oxidase 2 (SCO2), glutathione S-transferase theta 1 (GSTT1) and UDP glucuronosyltransferase family 1 member A3 (UGT1A3) were not detected and expressed in TCGA-COAD patients, so they were excluded. Finally, 479 genes related to copper metabolism were used for subsequent analysis. Clinical information in TCGA and GEO data sets included survival status, survival time, age, gender, TNM stage, lymph node metastasis and distant metastasis. Patients lacking corresponding clinical information were excluded from subsequent analysis.

### Analysis of differentially expressed genes

Limma package in R language was used to analyze the expression of 479 CMRGs in COAD patients. GEPIA2 (http://gepia2.cancer-pku.cn/#index) was used to analyze the expression of 479 genes in COAD patients. GEPIA2 database combines the RNA transcriptome data of normal tissue samples in GTEx database with TCGA data, enlarges the normal sample size, and is conducive to analyzing the expression of intergenes between normal and tumor tissues. The genes with the same differential expression trend were screened out by mutual verification between databases after expanding the sample size. Therefore, genes with the same expression trend in GEPIA2 and TCGA databases were screened out for subsequent analysis. The screening criteria for differential genes were p< 0.05 and | log2 fold change | ≥1.

### Gene mutation and immunoassay

The maftools package in R language was used to visually analyze the mutation frequency of patients’ mutated genes. Gsva packages in R language were used to calculate the score of infiltrating immune cells and evaluate the activity of immune-related pathways. Then the expression levels of differentially expressed CMRGs and the correlation between the expression patterns of CMRGs and immune cells and immune function were analyzed. TIMER database (https://cistrome.shinyapps.io/timer/) was used to reveal the correlation between CMRGs in risk model and tumor immune cells infiltrating in COAD.

### Protein-protein interaction network and gene function

The STRING database (https://string-db.org/) was used to analyze the interactions of differentially expressed copper metabolism-related gene proteins. The minimum required interaction score in STRING database is 0.4, which was also the default value in the database. Cytoscape software (Version 3.8.1) calculated the degree of interaction between differentially expressed CMRGs. CytoHubba is a plug-in in Cytoscape, which can discover key genes of complex networks according to Maximal Clique Centrality (MCC) algorithm. The greater the degree, the more interaction with other CMRGs, and the more important the gene was in CMRGs. GO and KEGG were used for the cellular functions and signaling pathways involved in the differentially expressed CMRGs, p<0.05 and q<0.05 was considered statistically significant.

### Consensus clustering of CMRGs

ConsensusClusterPlus package of R language was used for tumor classification analysis. 479 CMRGs were used as the basis for subtype analysis of COAD patients. The survival differences among COAD classifications were analyzed by K-M curve. Meanwhile, the differentially expressed genes based on CMRGs in COAD classification were analyzed, and these differentially expressed genes were used to classify tumors again by ConsensusClusterPlus package. Finally, the correlation between the two-tumor classification and tumor immune cell infiltration and immune function was analyzed to evaluate the value of tumor patient classification based on CMRGs for immunotherapy.

### Risk model construction

Univariate cox regression analysis was used to analyze the differentially expressed CMRGs. P< 0.05 was used as the standard to screen out CMRGs related to the prognosis of COAD patients. Then, multivariate cox regression analysis was used to further screen CMRGs related to the prognosis of COAD patients and construct a prognostic risk model of COAD patients. The patients were scored according to the prognostic risk model, and the optimal cut-off value was determined according to the software X-tile (12). Then, the patients were divided into high-risk patients and low-risk patients. The survival difference between high-risk patients and low-risk patients in TCGA and GSE39582 was analyzed by K-M curve.

### Nomogram construction

The rms package for R was used to build the nomogram. Nomogram was constructed based on clinical information (age and TNM stage) and tumor risk scores from TCGA and GSE39582 databases. At the same time, the calibration curve was used to analyze the consistency of nomogram in predicting the prognosis of patients with actual survival status.

### Tissue sample collection and ethics statement

A total of nineteen pairs of fresh tumor tissues from COAD patients and their paired normal tissues were collected from Ruijin Hospital of Shanghai Jiao Tong University, Shanghai Jiao Tong University School of Medicine, which was approved by the Human Research Ethics Committee of this hospital. Tumor samples were collected from patients with COAD and confirmed to be COAD in the pathological report, which maximally ensured that the collected samples were COAD samples containing tumor cells. In addition, all freshly paired samples collected from tumor patients were stored in a refrigerator at -80° C before we extracted RNA from tissue samples. The goal was to reduce the degradation of RNA in the tissue and ensure that RNA can be extracted from all samples.

### Quantitative real-time PCR

The total RNAs from tissues were extracted with the Trizol reagent (Servicebio, WUH, China). The NanoDrop 2000 spectrophotometer (Thermo) was used to quantify RNA, and cDNA was generated by the Servicebio^®^RT First Strand cDNA Synthesis Kit (Servicebio, WUH, China) and then analyzed using RT-qPCR with the SYBR Green qPCR Master Mix (High ROX) (Servicebio, WUH, China) on the 7,500 Fast Real-Time PCR System (Applied Biosystems, CA, USA). β-actin was exploited as an internal reference. The mRNA relative expression of individual genes was detected by 2^−ΔCt^ methods. The primer sequences used for analysis were listed in **Table 1**.

**Table 1:** Primer sequences of genes in the risk model used for qPCR.

Gene	Primer sequence (5’-3’)
β-actin	F:GACCTGTACGCCAACACAGT
	R:CTCAGGAGGAGCAATGATCT
CCND1	F:CCTCGGTGTCCTACTTCAAATGT
	R:ATGGAGTTGTCGGTGTAGATGC
CYP2S1	F:AGATGGCACAGGAGGAACAAA
	R:TTCATCAGGAGCAGGAGGGTA
FABP4	F:ACAGGAAAGTCAAGAGCACCAT
	R:ACGCATTCCACCACCAGTTT
GSTM1	F:GAAAAGAAGTACACGATGGGGGA
	R:AGTAGGGCAGATTGGGAAAGTC
MAPT	F:AAAAGCAAAGACGGGACTGGA
	R:TTCGGGAAGTGACAGAAGAGAC

qPCR, quantitative real-time polymerase chain reaction.

### Statistical analysis

R version 4.0.5 and Perl version 5.28 were used to perform statistical analysis. Excel office 2019 was used to organize data from TCGA and GEO database. P-value significant codes: 0≤ ****<0.0001≤ ***< 0.001 ≤ **< 0.01 ≤ *< 0.05 ≤.< 0.1.

## Results

### Research design process

**Figure 1:**
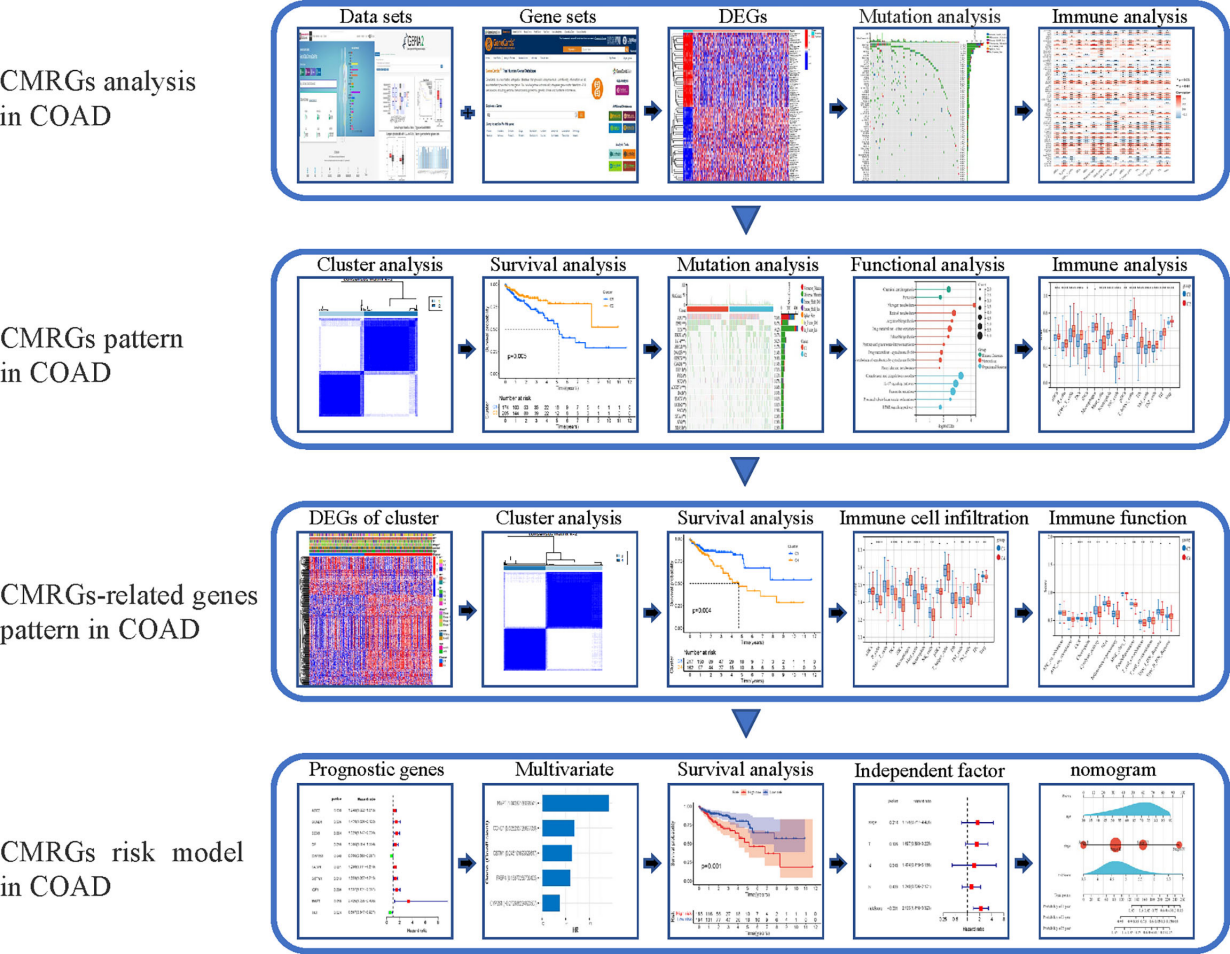
The workflow of research. CMRGs, Copper metabolization-related genes; COAD, Copper metabolization-related genes; DEG, differentially expressed gene.

In this study, the expression of 479 CMRGs downloaded from GeneCards was analyzed using TCGA and GEPIA2 databases. Then, mutations, immune characteristics, participating functions and signaling pathways of differentially expressed CMRGs in COAD were systematically analyzed. In addition, the COAD patients were classified based on 479 CMRGs, and further classified according to the differentially expressed CMRGs related genes among different categories. The two types of COAD patients were analyzed from the aspects of immune characteristics, participating functions, signaling pathways, survival differences, etc. Finally, risk model and nomogram for COAD patients was constructed to analyze the prognostic value of CMRGs. The flow chart of the entire research process is shown in **Figure 1**.

### Differentially expressed genes related to copper metabolism

**Figure 2:**
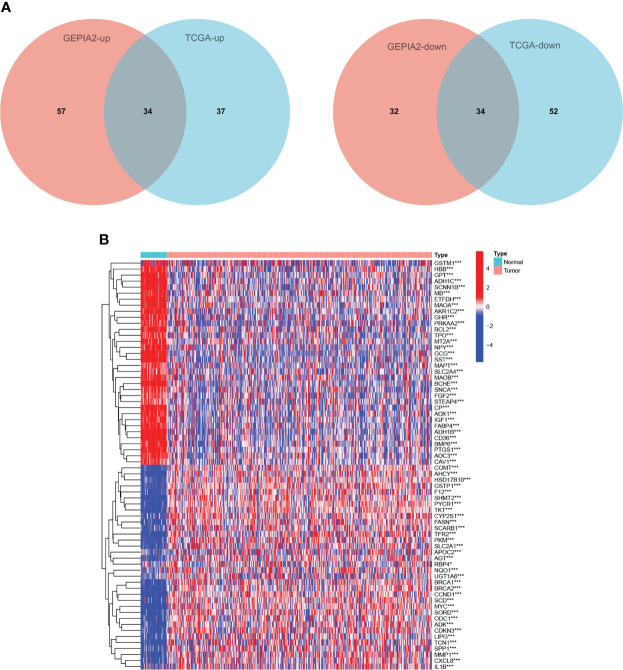
Differentially expressed CMRGs. **(A)** Venn diagram of differential expression of 479 CMRGs in tumor tissues from GEPIA2 and TCGA databases. **(B)** Heat map of 68 differentially expressed genes in TCGA, Red represents up-regulated expression, blue represents down-regulated expression. TCGA, The Cancer Genome Atlas; GEPIA2, Gene Expression Profiling Interactive Analysis.

In GEPIA2 database, 157 of 479 CMRGs were differentially expressed between tumor tissues and normal tissues in COAD patients, among which 91 genes were up-regulated and 66 genes were down-regulated in tumors. In TCGA database, 157 of 479 CMRGs were differentially expressed between tumor tissues and normal tissues in COAD patients, among which 71 genes were up-regulated and 86 genes were down-regulated in tumors (**Supplementary Figure S1**). Genes that were up-regulated or down-regulated in GEPIA2 and TCGA data were screened out. As shown in **Figure 2A**, 34 CMRGs were up-regulated and 34 CMRGs were down-regulated in tumor tissues in the two databases. **Figure 2B** visually shows 34 up-regulated genes and 34 down-regulated genes in the form of heat maps.

### Protein interactions and mutations of CMRGs

**Figure 3:**
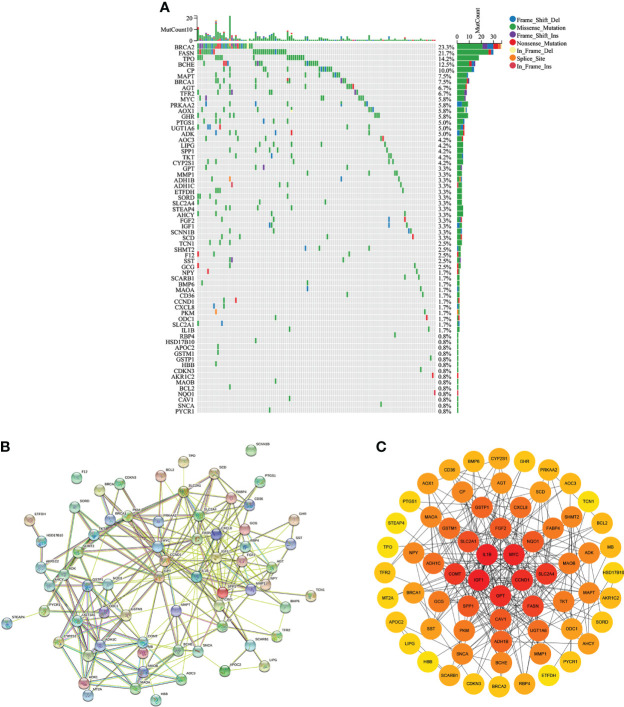
Gene mutation and protein interaction. **(A)** Mutations of differentially expressed CMRGs in COAD tumors. **(B)** Protein interactions of differentially expressed genes in STRING database. **(C)** Cytoscape software analyzed the degree of interaction between differentially expressed CMRGs.

As shown in **Figure 3A**, 64 differentially expressed CMRGs were mutated in tumor tissues of COAD patients, with the highest detected mutation reaching 23.3% and the lowest detected mutation reaching 0.8%. No mutations in catechol-O-methyltransferase (COMT), fatty acid binding protein 4 (FABP4), myoglobin (MB) and metallothionein 2A (MT2A) were detected in COAD tumor tissues. STRING database analysis of 68 differentially expressed copper metabolism genes showed that the number of nodes was 68, the number of edges was 254, the PPI enrichment p-value<0.05 (**Figure 3B**). CytoScape analysis showed that CMRGs had a wide range of interactions, which jointly participated in the regulation process of the body and affected the progression of tumor. Among them, interleukin 1, beta (IL1B), v-myc avian myelocytomatosis viral oncogene homolog (MYC), cyclin D1 (CCND1), glutamic-pyruvate transaminase (GPT) and insulin-like growth factor 1(IGF1) played an important role in CMRGs and had more interactions with other genes (**Figure 3C**).

### Functional and immune characteristics analysis of CMRGs

**Figure 4:**
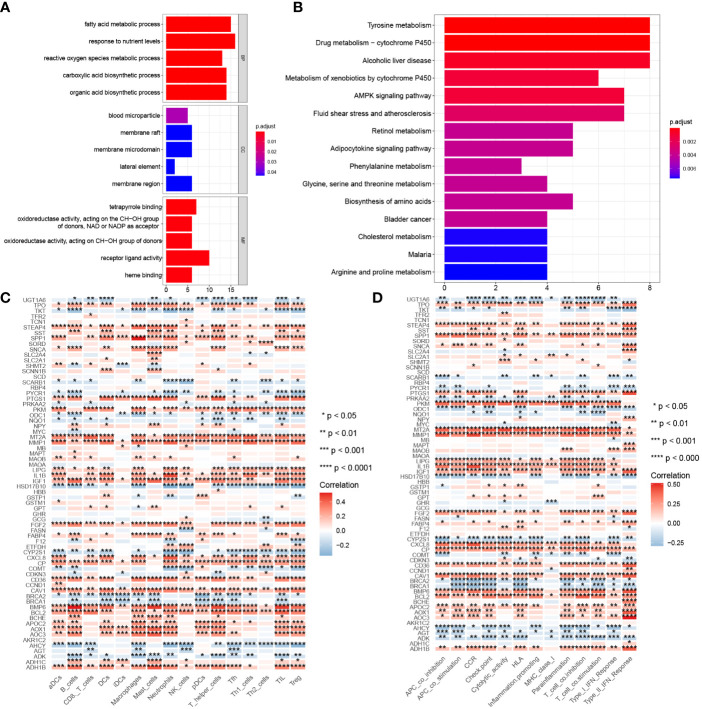
Gene function and correlation with immunity. **(A)** GO analysis of 68 differentially expressed CMRGs. **(B)** KEGG analysis of 68 differentially expressed CMRGs. **(C)** Correlation between 68 differentially expressed CMRGs and immune cell infiltration in tumors. **(D)** Correlation between 68 differentially expressed CMRGs and immune function in tumors. GO, Gene Ontology; BP, biological process; CC, cellular component; MF, molecular function; KEGG, Kyoto Encyclopedia of Genes and Genomes. 0≤ ****<0 .0001≤ *** < 0.001 ≤ ** < 0.01 ≤ * < 0.05 ≤. < 0.1.

GO analysis in **Figure 4A** show that, 68 differentially expressed CMRGs were involved in biological processes (BP) such as fatty acid metabolic process, response to nutrient levels and reactive oxygen species metabolic process, cellular component (CC) such as blood microparticle, membrane raft, membrane microdomain, molecular function (MF) such as receptor ligand activity, receptor ligand activity, heme binding. KEGG analysis in **Figure 4B** showed that 68 differentially expressed CMRGs were involved in the conduction process of tyrosine metabolism and drug metabolism-cytochrome P450 signaling pathways. Analysis of tumor immune cell infiltration and immune function in COAD patients showed (**Figures 4C, D**) that CMRGs were positively correlated with the immune cells’ infiltration and immune function in tumors. The higher the expression of related genes, the more the infiltration of related immune cells.

### Differential immune characteristics of CMRGs pattern

**Figure 5:**
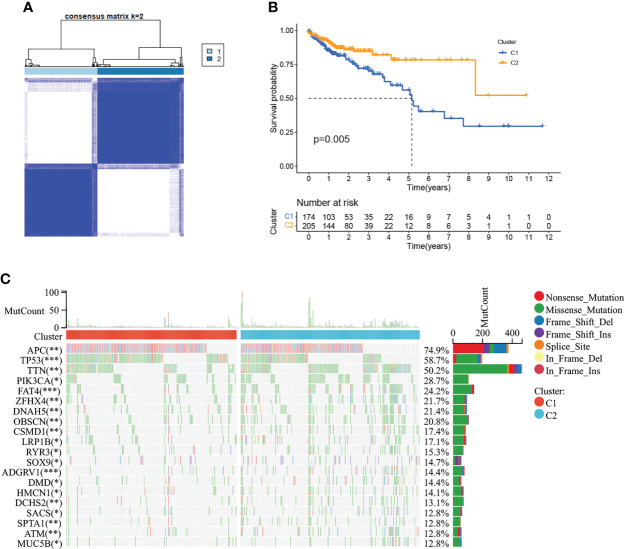
Tumor classification based on the CMRGs. **(A)** COAD patients were grouped into two clusters according to the consensus clustering matrix (k = 2). **(B)** Kaplan–Meier curves for the two clusters. **(C)** The top 20 genes with the highest degree of mutation between C1 and C2. 0≤ ****<0 .0001≤ *** < 0.001 ≤ ** < 0.01 ≤ * < 0.05 ≤. < 0.1.

**Figure 6:**
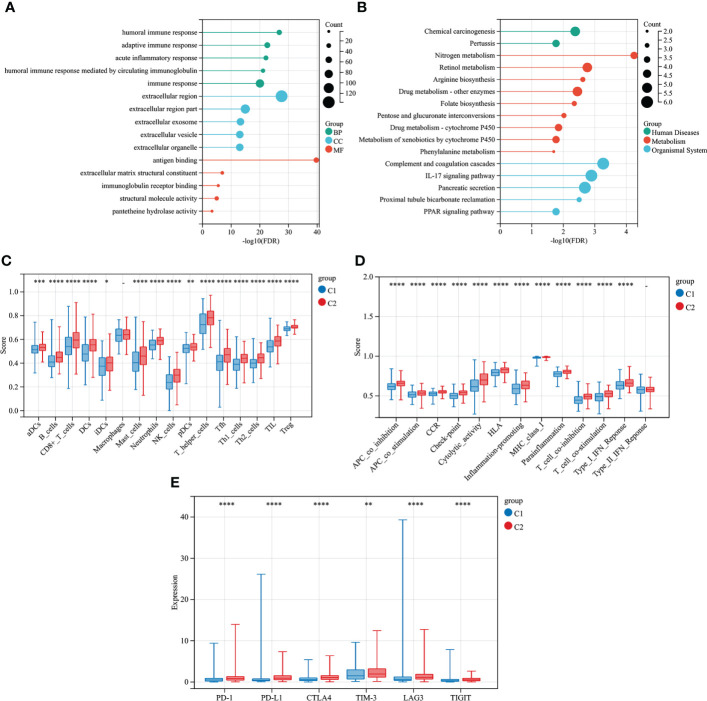
Differential immune characteristics of CMRGs pattern C1 and C2. **(A)** GO Lollipop graph for genes in BP, CC, and MF. **(B)** Lollipop graph of KEGG pathways with the most enriched genes; the vertical axis refers to names of the pathway; and the horizontal axis refers to the number of genes. **(C)** Relative infiltration of 16 types of immune cells in CMRGs cluster C1 and C2. **(D)** Relative enrichment score of 13 immune related functions in CMRGs cluster C1 and C2. **(E)** Differences in expression of immune checkpoint - related genes between C1 and C2. 0≤ ****<0 .0001≤ *** < 0.001 ≤ ** < 0.01 ≤ * < 0.05 ≤. < 0.1.

479 CMRGs were used to subtype COAD patients, and the results suggested (**Figure 5A**) that patients could be divided into C1 and C2 subtypes. The prognostic analysis of C1 and C2 subtypes showed that the prognosis of C1 subtype was worse than that of C2 subtype (**Figure 5B**, P =0.005). Meanwhile, according to p< 0.05, the differences in CMRGs expression between C1 and C2 subtypes were analyzed, and the results showed (**Supplementary Figure S2A**) that most CMRGs were up-regulated in C2 subtypes. Analysis results of mutated genes between C1 and C2 showed (**Figure 5C**) that the mutation frequency of C1 subtype was higher than that of C2 subtype, especially mutations of tumor suppressor genes such as APC and TP53 that play a role in the occurrence and development of COAD. In **Supplementary Figure S2B**, differentially expressed genes and clinical features between C1 and C2 subtypes were analyzed, and the results showed that lymph node metastasis, distant metastasis, *in situ* invasion and TNM staging of C1 subtype were more severe than C2 subtype. In addition, most of the 348 differentially expressed genes were up-regulated in C2 subtype. **Figures 6A, B** shows the cell functions and cell signaling pathways involved in the differentially expressed genes of C1 and C2. The results showed that the differentially expressed genes between C1 and C2 were involved in a variety of immune functions and metabolic signaling processes in cells. In **Figures 6C, D** the analysis results of immune characteristics between C1 and C2 subtypes showed that C2 subtype with a better prognosis had more infiltrated immune cells and higher immune function activation. In **Figure 6E**, C2 patients had higher levels of immune checkpoint related gene transcription, suggesting that C2 patients could benefit from immunotherapy.

### Differential immune characteristics of CMRGs-related genes pattern

**Figure 7:**
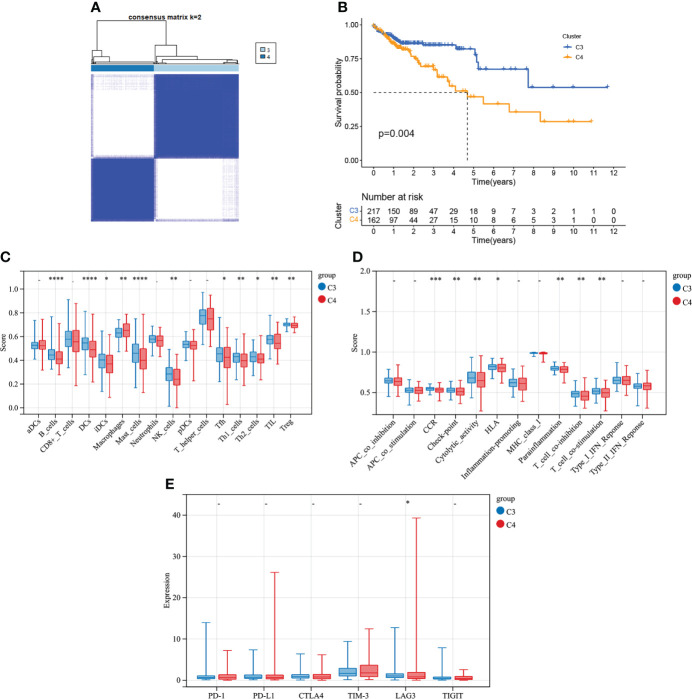
Tumor classification based on the CMRGs-related gene. **(A)** COAD patients were grouped into two clusters according to the consensus clustering matrix (k = 2). **(B)** Kaplan–Meier curves for the two clusters. **(C)** Relative infiltration of 16 types of immune cells in CMRGs-related gene cluster C3 and C4. **(D)** Relative enrichment score of 13 immune related functions in CMRGs-related gene cluster C3 and C4. **(E)** Differences in expression of immune checkpoint - related genes between C3 and C4. 0≤ ****<0 .0001≤ *** < 0.001 ≤ ** < 0.01 ≤ * < 0.05 ≤. < 0.1.

COAD was divided into two types by 348 CMRGs-related genes (**Figure 7A**). There was a difference in prognosis between the two types (C3 and C4), with C3 having a better prognosis and the difference being statistically significant (**Figure 7B**). At the same time, the analysis of immune characteristics between C3 and C4 showed (**Figures 7C–E**) that the level of immune cell infiltration and immune function activation of C3 tumor with better prognosis was higher than that of C4, and the expression level of C3 immune checkpoint related gene lymphocyte-activation gene 3 (LAG3) was higher, suggesting that C3 tumor was more suitable for immunotherapy. These results suggested that further refinement of subtypes C1 and C2 for C3 and C4 may help to identify patients with cancer who may benefit from clinical immunotherapy.

### Risk model construction

In **Supplementary Figure S3A**, 10 genes related to the prognosis of COAD patients were screened out from 68 differently-expressed genes related to copper metabolism by univariate regression analysis, among which 8 genes, amine oxidase, copper containing 3 (AOC3), CCND1, CD36, ceruloplasmin (CP), FABP4, glutathione S-transferase mu 1 (GSTM1), IGF1 and microtubule-associated protein tau (MAPT), were prognostic risk factors. cytochrome P450, family 2, subfamily S, polypeptide 1 (CYP2S1) and transketolase (TKT) were prognostic protective factors. In **Supplementary Figure S3B**, multivariate regression analysis was used to further screen the 10 genes, and finally it was determined that CCND1, CYP2S1, FABP4, GSTM1 and MAPT were used to construct the risk model. Akaike Information Criterion (AIC) was 712.18. The calculation formula of the risk model was as follows:


risk Score=CCND1*0.326233727+ CYP2S1*−0.272986225+FABP4*0.188772687+GSTM1*0.245101694+MAPT*1.042871661

**Figure 8:**
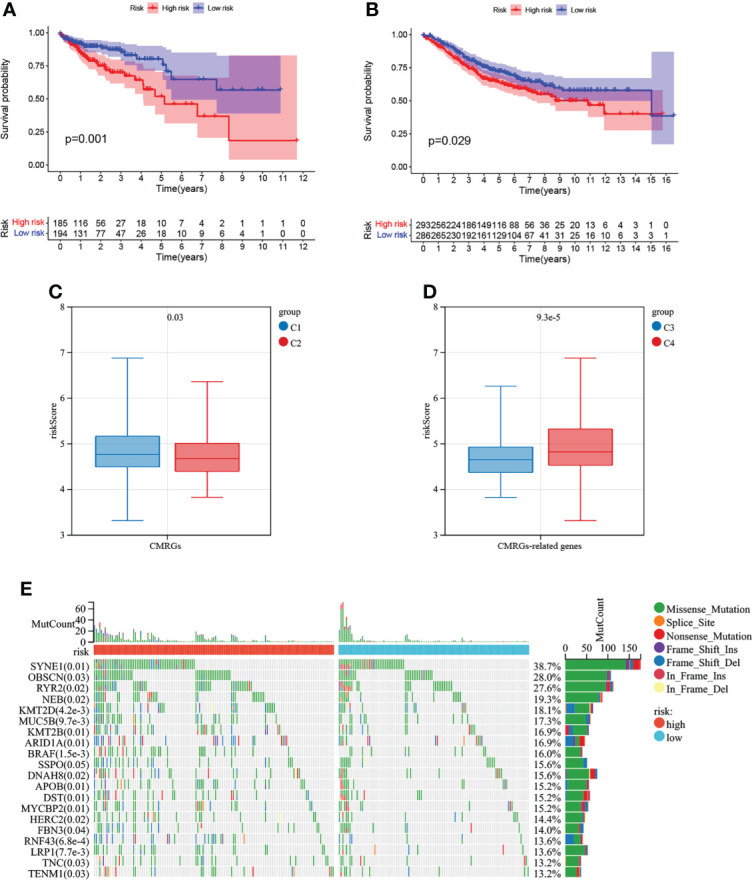
Survival analysis of risk models and correlation with COAD subtypes. **(A)** Analysis of survival differences between high-risk and low-risk COAD patients in TCGA database. **(B)** Analysis of survival differences between high-risk and low-risk COAD patients in GSE39582. **(C)**The correlation of risk score with CMRGs pattern. **(D)** The correlation of risk score with CMRGs-related genes pattern. **(E)** Genetic mutations between high and low risk groups.

The risk score of each COAD patient in TCGA and GEO database was calculated according to the risk model, and 4.705 was obtained by X-tile software as a cutoff value to divide patients into high-risk patients and low-risk patients. The results of K-M curve suggested (**Figures 8A, B**) that the risk model constructed by CMRGs had a difference in prognosis between patients with high and low risk, and patients with high risk had a poor prognosis. **Supplementary Figure S4A** visually showed the distribution of risk among patients, and **Supplementary Figure S4B** visually showed that high-risk patients had a higher mortality rate than low-risk patients. In **Supplementary Figures S4C,D**, univariate and multivariate cox regression analysis suggested that this risk model was an independent prognostic risk factor for COAD patients. In **Supplementary Figure S4E**, the ROC curve showed that the risk model based on CMRGs can be used to predict the prognosis of cancer patients.

The results of **Figures 8C, D** analysis showed that the risk score of this risk model was different not only between C1 and C2 subtypes, but also between C3 and C4 subtypes, indicating that this risk model could be associated with C1 and C2 subtypes as well as C3 and C4 subtypes, simplifying the steps of COAD subtype identification and contributing to the identification of tumor patients suitable for immunotherapy. The top 20 genes with mutation differences between patients at high and low risk are shown in **Figure 8E**. Patients in the high-risk group have higher frequency of mutation than patients at low risk. The expression and clinical characteristics of 5 model genes between the high-low risk groups were shown in **Supplementary Figure S5A**. In **Supplementary Figure S5B**, the correlation between CMRGs used for model construction and tumor immune cell infiltration in COAD patients was listed, suggesting that the expression of these genes may be involved in the formation of tumor immune microenvironment

### Nomogram construction

**Figure 9:**
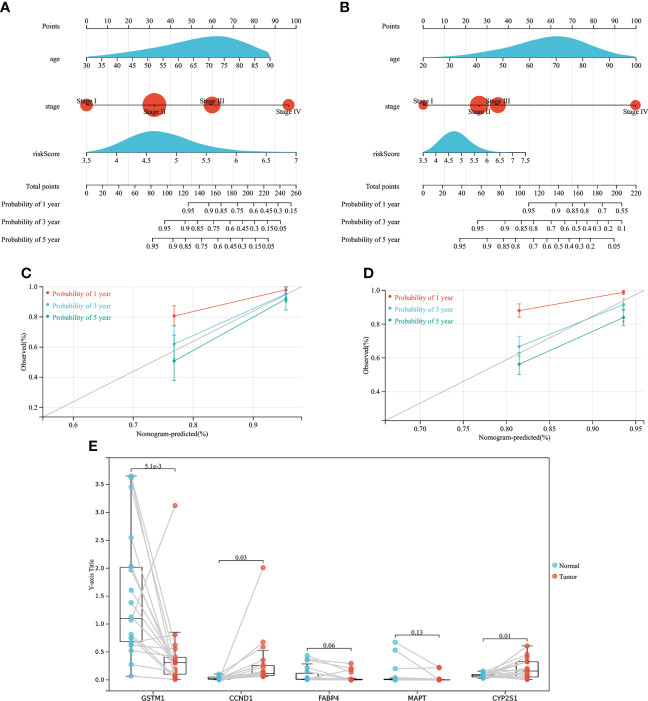
The establishment of the nomogram and gene expression validation. **(A)** Establish a Nomogram based on TCGA. **(B)** Establish a Nomogram based on GEO. **(C)** 1-, 3-, 5-year calibration curves of TCGA. **(D)** 1-, 3-, 5-year calibration curves of GEO. **(E)** Gene expression validation.

As shown in **Figure 9A**, a nomogram based on age, TNM staging and risk model was constructed using TCGA database with a C-index of 0.797. As shown in **Figure 9B**, a nomogram based on age, TNM staging and risk model was constructed using GSE39582 data set with a C-index of 0.716. Meanwhile, calibration curves of TCGA and GSE39582 were drawn in **Figures 9C, D** respectively. The results suggested that nomogram based on the risk score of CMRGs had little difference from the actual situation in predicting the prognosis of patients with COAD, and can be used well for predicting the prognosis of patients with COAD.

### The mRNA relative expression of five genes

As showed in **Figure 9E**, mRNA expression levels of genes associated with risk models were measured in samples from COAD patients. The mRNA expression of CCND1 and CYP2S1 genes was high in COAD tumor tissues, while the mRNA expression of GSTM1 gene was low in COAD tumor tissues, and the difference was statistically significant, which was consistent with the previous results of TCGA database and GEPIA2 data analysis. However, there was no statistical difference in the mRNA expression of FABP4 and MAPT between tumor tissues and normal tissues of COAD patients, but the downward trend of FABP4 and MAPT expression in tumor tissues could still be seen, which may need to be verified by more clinical samples. In conclusion, the clinical samples of COAD patients demonstrated that the genes used to construct the risk model were expressed differently between normal tissues and tumor tissues, which further verified that the genes screened by TCGA and GEPIA2 databases were in line with the sample validation results.

## Discussion

In this study, COAD subtypes and risk models were constructed based on CMRGs. Meanwhile, the association of genes, subtypes, risk model with the immune cells and immune function were analyzed. The results proved that the CMRGs not only can distinguish between clinical characteristics and immune characteristic differences of COAD subtypes, and prognostic risk model can also be used to predict the prognosis of patients with cancer. This suggested that CMRGs played an important role in predicting the prognosis of COAD patients, helping to identify patients who were eligible for immunotherapy, and providing new biomolecular targets for COAD treatment.

Abnormal levels of copper can lead to inflammation and cancer (13). Copper can be involved in angiogenesis, tumor growth and metastasis, and cancer cells can adapt to adverse microenvironment by regulating copper metabolism (14, 15). In triple negative breast cancer, the decrease of mitochondrial copper content can reduce the energy production of tumor cells, thus promoting the apoptosis of tumor (16). In ovarian cancer and non-small cell lung cancer, the activity and expression of copper-associated transporters are involved in the formation of tumor resistance to cisplatin and other platinum compounds (14). In COAD, the formation of KRAS-induced tumor and the generation of drug resistance are related to copper metabolism, and the use of copper chelating agent can inhibit the growth of tumor cells (17). In addition, copper-associated metabolic proteins can enhance the sensitivity of COAD cell lines to oxaliplatin (18). At the same time, many studies have shown that copper chelating agent has anti-tumor and anti-metastasis effects (19). These studies suggested that copper metabolism plays an important role in tumors.

CMRGs refers to the genes involved in intracellular copper metabolism, including not only the genes encoding copper-containing proteins and regulating copper transporters, but also the related genes regulating the synthesis of these proteins and other functional processes directly or indirectly regulating copper in cells. For example, in risk model, CCND1 was the major regulator of the cell cycle, and its transcription factor participate in copper excretion (20, 21). In addition, copper is also involved in cell cycle regulation (22). These studies suggested that genes in our research associated with copper metabolism directly or indirectly, which can reduce or increase the content of copper in cell. Meanwhile, five CMRGs, CCND1, CYP2S1, FABP4, GSTM1 and MAPT, which were used to construct risk models, had been studied in COAD. Activation of CCND1 can promote the proliferation and invasion of COAD cells, while inhibition of CCND1 expression and activity can induce cell cycle arrest of COAD cells (23–25). Promoting the expression of CYP2S1 can enhance the effect of oxaliplatin on COAD cells, while knocking down CYP2S1 can promote the expression of prostaglandin E2, thus promoting tumor proliferation (26, 27). FABP4 is highly expressed in COAD and correlated with lymph node metastasis, TNM stage and tumor differentiation (28). GSTM1 polymorphism is associated with COAD susceptibility in different ethnic groups (29, 30). Methylation of the MAPT gene is associated with poor prognosis in COAD (31). In addition, in this study, clinical samples were used to prove the expression of related genes in the risk model. Among them, CCND1, GSTM1 and CYP2S1 were differentially expressed in normal tissues and tumor tissues, while the other two genes had the same expression trend as the database analysis. These studies and the results verified in this study together prove that CMRGs used to construct risk models are correlated with COAD.

Cancer immune escape is a hallmark of tumor development. Inorganic copper can directly enter the blood and participate in immunosuppression (22). Increased copper content can promote the expression of PD-L1 in tumors and drive the immune escape of tumors, while decreased copper content can increase the infiltration of CD8+T cells and natural killer cells (32). These studies suggested that intracellular copper metabolism affected the change of copper content in tumor immune regulation and the formation of immune microenvironment. Immune cell infiltration is essential for immunotherapy (33). In this research, the expression of most CMRGs was positively correlated with immune cells infiltration, suggesting that these genes might regulate tumor immune cell invasion by regulating copper content. Therefore, identification of patients with high CMRGs expression can allow patients to benefit from immunotherapy, while patients with low immunotherapy benefit can be assisted to improve the sensitivity of patients to immunotherapy by regulating the expression of CMRGs. This was further verified when we analyzed COAD subtypes using CMRGs. Patients with high CMRGs expression had a significantly better prognosis than those with low CMRGs expression. Meanwhile, the expression of immune checkpoint related genes in subtype patients with high expression of CMRGs was significantly increased, indicating that patients with high expression of CMRGs were more suitable for immunotherapy. All these indicated that there was an important interaction between CMRGs and tumor immunity. Studying the effect of CMRGs on tumor copper metabolism will contribute to tumor immunotherapy.

In this study, CMRGs were used for COAD typing, which could distinguish patients with different clinical outcomes and characteristics. At the same time, the risk model constructed can be well correlated with typing, which is helpful to simplify the gene measurement required for typing and can also be used to predict the prognosis of patients. However, there were still some shortcomings. First, more clinical data were needed to validate the COAD typing and risk model. The second was that the mechanism by which the genes used to build the model work in COAD was unclear, which needs to be further studied in future studies

## Conclusions

CMRGs were associated with immune infiltration of COAD. COAD subtypes can be well analyzed by CMRGs, to distinguish tumor patients with different clinical and immune characteristics. In addition, this risk model and nomogram based on CMRGs can be used to predict the prognosis of tumor patients, and the risk model was related to subtypes, which will simplify the clinical identification process of copper metabolization-related subtypes and better identify patients who can benefit from copper metabolization-related therapy and immunotherapy

## Data availability statement

The datasets presented in this study can be found in online repositories. The names of the repository/repositories and accession number(s) can be found in the article/supplementary material.

## Ethics statement

The studies involving human participants were reviewed and approved by the Human Research Ethics Committee of Ruijin Hospital of Shanghai Jiao Tong University. The patients/participants provided their written informed consent to participate in this study.

## Author contributions

The article was written by JL. BL and XY contributed equally to this work. WC, MW and ZY have provided guidance to the manuscript preparation. All authors have approved the final version of the editorial.

## Funding

This work was supported by the Shanghai Municipal Science and Technology Commission (19441905400); and Shanghai Jiaotong University (YG2019ZDA15); and Shanghai Municipal Commission of Health and Family Planning (No. 2017-239).

## Acknowledgments

We thank all the authors who contributed to this topic. And thanks to the TCGA and GEO databases for providing data.

## Conflict of interest

The authors declare that the research was conducted in the absence of any commercial or financial relationships that could be construed as a potential conflict of interest.

## Publisher’s note

All claims expressed in this article are solely those of the authors and do not necessarily represent those of their affiliated organizations, or those of the publisher, the editors and the reviewers. Any product that may be evaluated in this article, or claim that may be made by its manufacturer, is not guaranteed or endorsed by the publisher.
