# Supplementary material for: Copper metabolism patterns and tumor microenvironment characterization in colon adenocarcinoma
Source: Front Oncol. 2022 Sep 20;12:959273. doi: 10.3389/fonc.2022.959273 (PMC9530986; doi:10.3389/fonc.2022.959273)
Supplement: Supplementary file 2 [file DataSheet_1.docx]

**Supplementary Information for**

**Copper Metabolism Patterns and Tumor Microenvironment Characterization in Colon Adenocarcinoma**

**Jianwei Lin^1†^,** **Bixian Luo^1†^, Xinbo Yu^2†^, Zheyu Yang^1*^, Mingliang Wang^1,3*^, Wei Cai^1*^**

^1^Department of General Surgery, Ruijin Hospital, Shanghai Jiao Tong University School of Medicine. Shanghai, Shanghai, China

^2^ Department of Urology, Ruijin Hospital, Shanghai Jiao Tong University School of Medicine. Shanghai, Shanghai, China

^3^Department of General Surgery, Ruijin Hospital Luwan Branch, Shanghai Jiao Tong University School of Medicine. Shanghai, Shanghai, China

Corresponding Author:

Wei Cai

Email address: [caiwei@shsmu.edu.cn](mailto:caiwei@shsmu.edu.cn)

Mingliang Wang

Email address: [wml_2902@163.com](mailto:wml_2902@163.com)

Zheyu Yang

Email address: [yongsmith@163.com](mailto:yongsmith@163.com)

†These authors have contributed equally to this work

Keywords: copper metabolism _1_, microenvironment _2_, colon adenocarcinoma _3_, riskscore_4_, nomogram_5_

This file includes:

Figure S1-S5


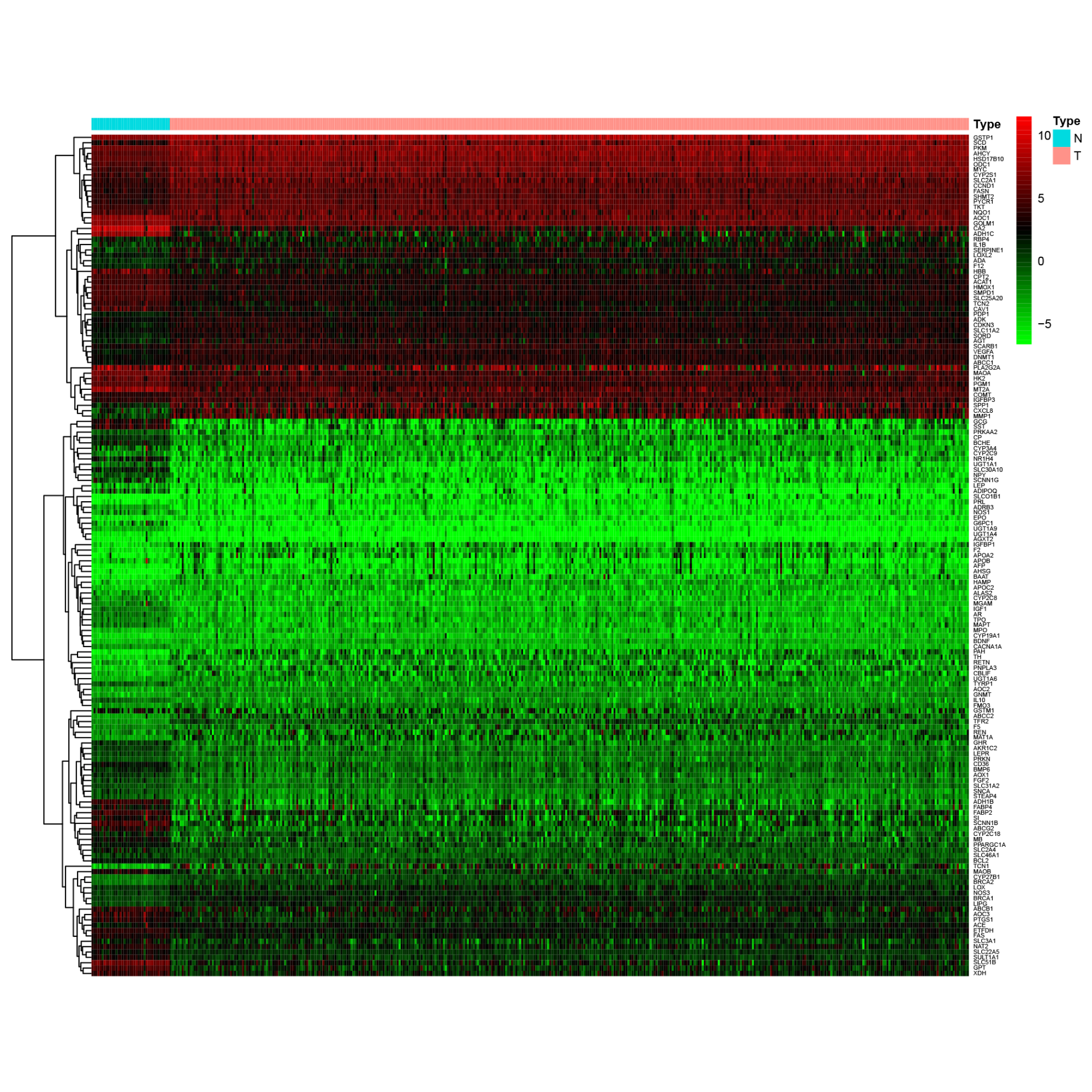


**Supplementary Figure S1.** Heatmap of differentially expressed CMRGs in TCGA, with green representing down-regulation and red representing up-regulation.


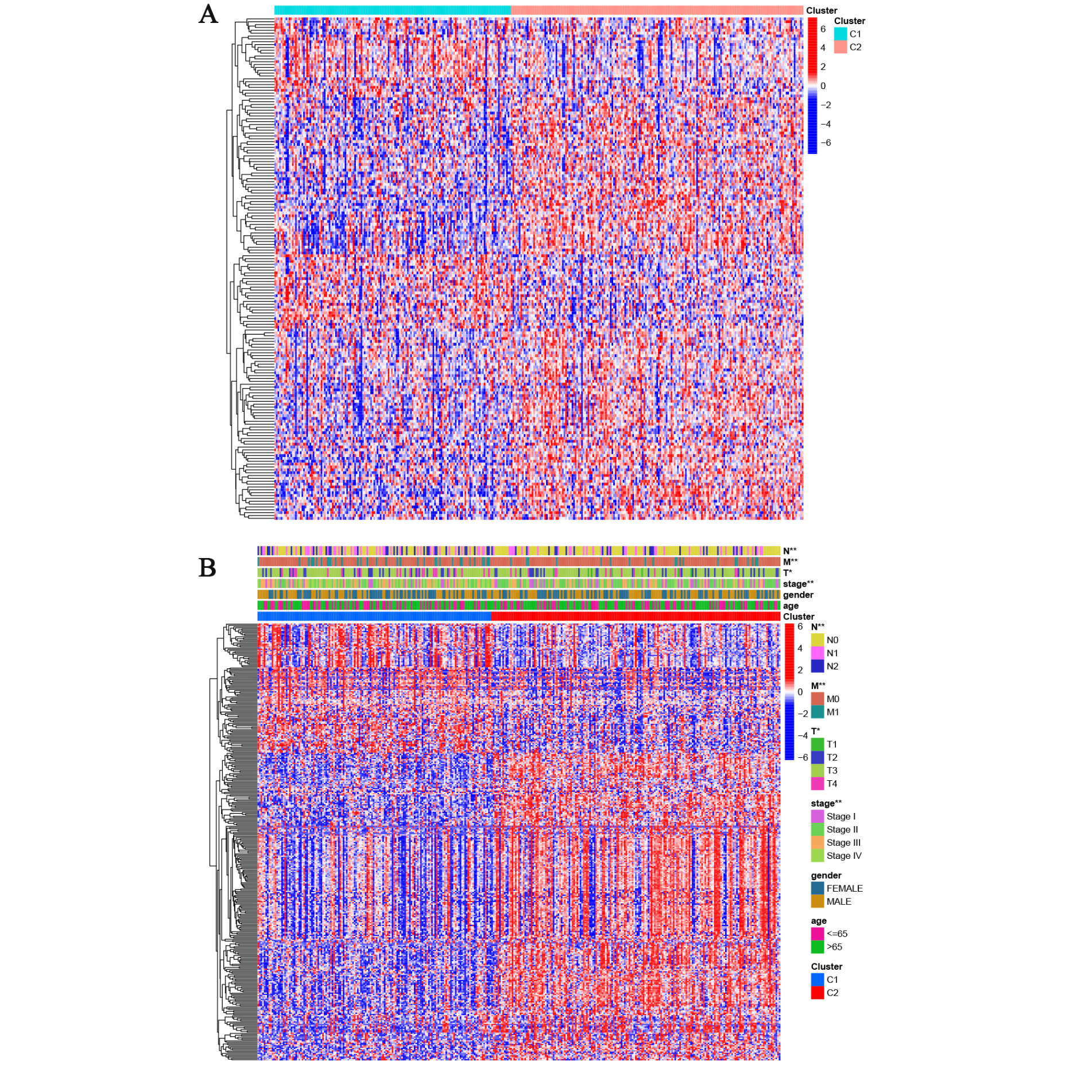


**Supplementary Figure S2.** Differential gene expression between C1 and C2. (A) Heatmap of differentially expressed CMRGs between C1 and C2, with blue representing down-regulation and red representing up-regulation. (B) Heatmap of differentially expressed CMRGs-related genes between C1 and C2, with blue representing down-regulation and red representing up-regulation.


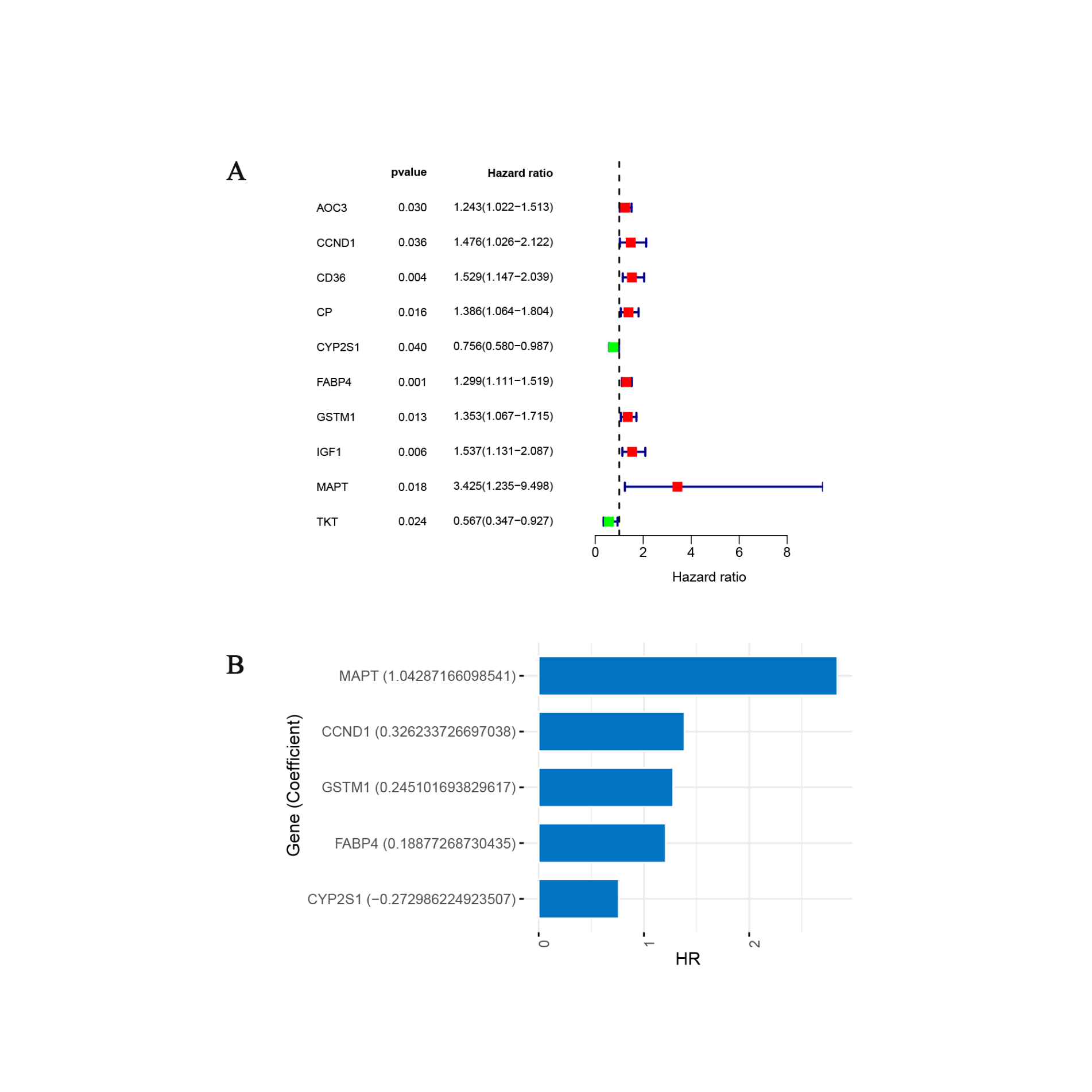


**Supplementary Figure S3.** Risk model construction. (A) Univariate cox regression analysis of differential expression of 68 CMRGs. (B) Multivariate cox regression analysis analyzed 10 genes in univariate cox regression analysis.


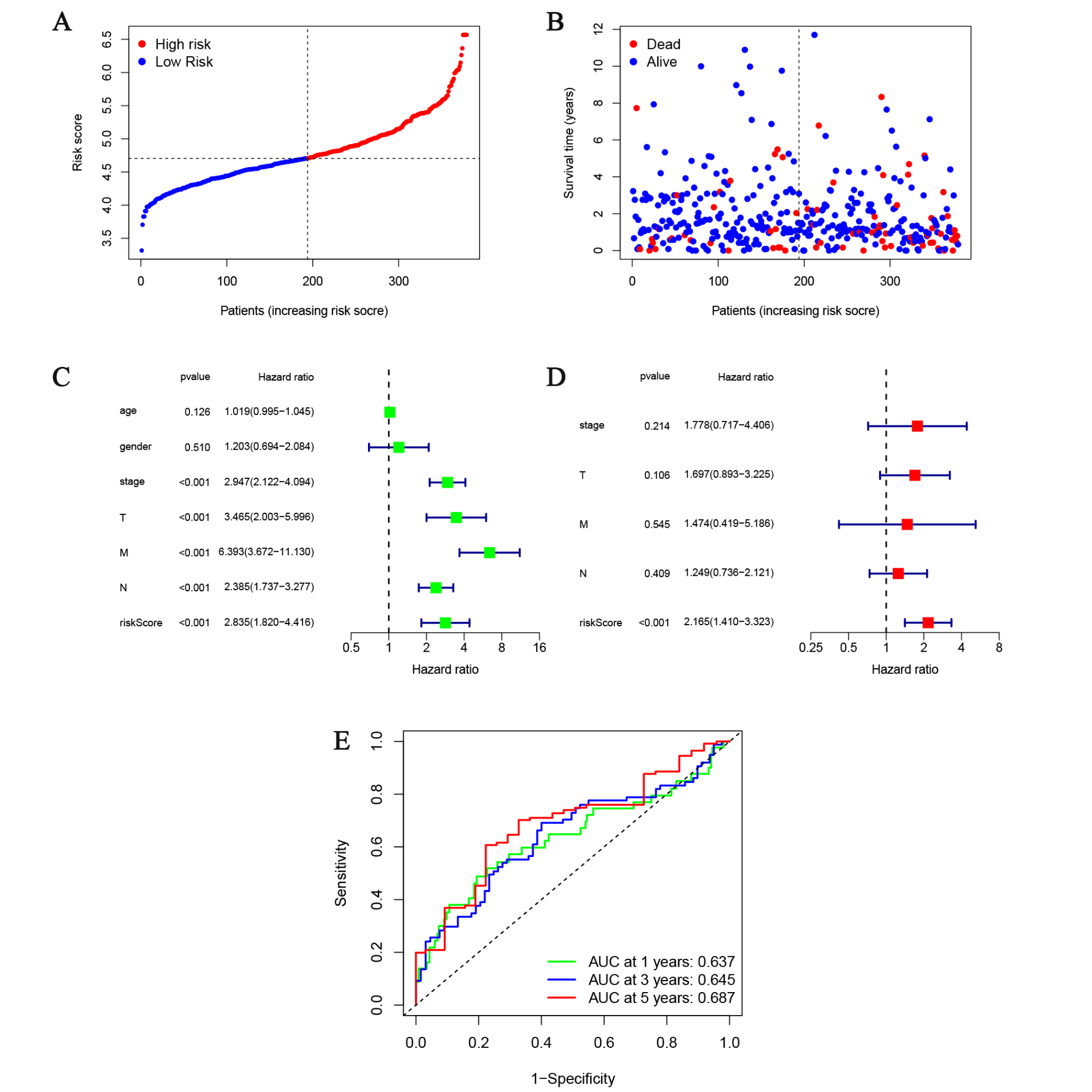


**Supplementary Figure S4.** Validation of risk model. (A) Distribution of risk scores of each COAD patient. (B) correlation between survival time and survival status of each patient. (C) Univariate cox regression analysis of factors associated with COAD prognosis. (D) Multivariate cox regression analysis of independent prognostic risk factors for COAD. (E) ROC curve analysis of risk model.


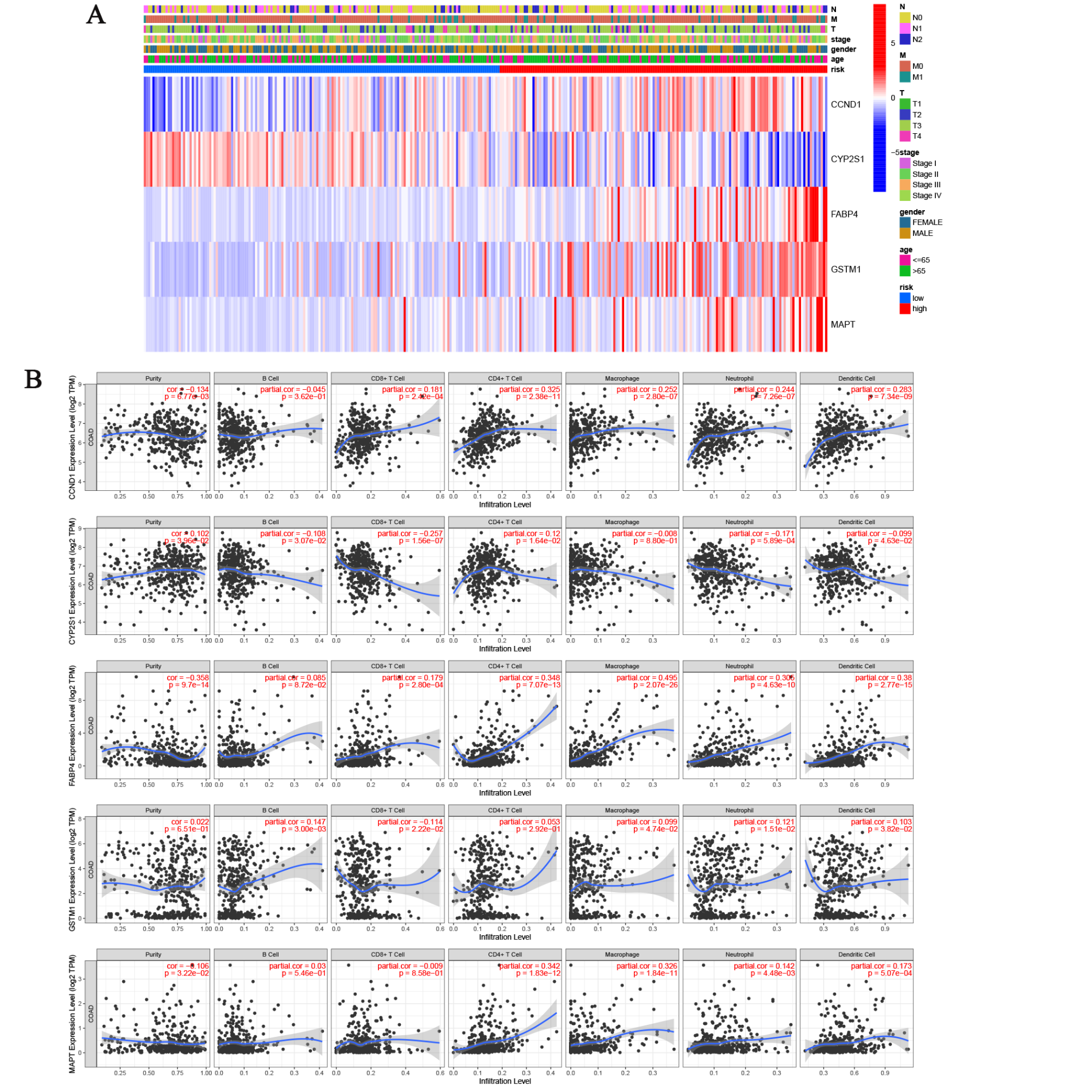


**Supplementary Figure S5.** Genetic immunity correlates in risk models. (A) The expression pattern of 5 CMRGs genes. (B) correlation between survival time and survival status of each patient. TIMER database was used to analyze the correlation between 5 CMRGs and immune cells.
